# Interleukin-8 produced from cancer-associated fibroblasts suppresses proliferation of the OCUCh-LM1 cancer cell line

**DOI:** 10.1186/s12885-022-09847-z

**Published:** 2022-07-08

**Authors:** Ryota Tanaka, Kenjiro Kimura, Shimpei Eguchi, Go Ohira, Shogo Tanaka, Ryosuke Amano, Hiroaki Tanaka, Masakazu Yashiro, Masaichi Ohira, Shoji Kubo

**Affiliations:** 1 Department of Hepato-Biliary-Pancreatic Surgery, Osaka City University Graduate School of Medicine, 1-4-3 Asahimachi, Abenoku, Osaka, 545-8585 Japan; 2 Department of Gastroenterological Surgery, Osaka City University Graduate School of Medicine, Osaka, Japan; 3 Department of Medical Oncology, Thomas Jefferson University, Philadelphia, PA USA; 4 Molecular Oncology and Therapeutics, Osaka City University Graduate School of Medicine, Osaka, Japan; 5 Cancer Center for Translational Research, Osaka City University Graduate School of Medicine, Osaka, Japan

**Keywords:** Bile duct cancers, Cancer-associated fibroblast, Interleukin-8, Pancreatic ductal adenocarcinoma, Suppressive CAFs

## Abstract

**Background:**

Cancer-associated fibroblasts (CAFs) play an important role in cancer growth by interacting with cancer cells, but their effects differ depending on the type of cancer. This study investigated the role of CAFs in biliary tract cancers (BTCs), compared with pancreatic ductal adenocarcinoma (PDAC) as a comparison cohort.

**Methods:**

We retrospectively evaluated alpha-smooth muscle actin (αSMA) expression in CAFs from 114 cases of PDAC and 154 cases of BTCs who underwent surgical treatment at our institution from 1996 to 2017. CAFs were isolated from resected specimens of BTC and PDAC, and tested for the effects of their supernatants and cytokines on cancer cell proliferation.

**Results:**

PDAC patients with positive αSMA expression showed significantly shorter overall survival and recurrence-free survival than αSMA-negative patients (*p* = 0.003, *p* = 0.009, respectively). BTC patients with positive αSMA expression showed better recurrence-free survival than αSMA-negative patients (*p* = 0.03). CAF-conditioned medium suppressed the proliferation of cancer cells for only OCUCh-LM1 cells and not PDAC cells. Blockage of Interleukin-8 (IL-8) or its receptor C-X-C motif chemokine receptor 2 (CXCR2) by antibodies canceled the suppressive effect of the IL-8.

**Conclusions:**

CAFs are a good prognostic factor in BTC, but not for PDAC. Moreover, CAF-produced Interleukin-8 suppresses the proliferation of OCUCh-LM1 cell lines.

**Supplementary Information:**

The online version contains supplementary material available at 10.1186/s12885-022-09847-z.

## Background

Biliary tract cancers (BTCs) are aggressive, and each has a poor prognosis. Despite advance in surgical treatments and chemotherapy that target cancer cells, their effects are limited. Recently, therapies targeting non-cancerous cells have been developed [1]. However, there are few reports on therapy that focus on tumor microenvironment, such as those of fibroblast in BTCs [2].

The stroma in solid tumors is composed of a rich extracellular matrix and many types of non-cancerous cells, including fibroblasts, myeloid cells, and lymphocytes, which play important roles in cancer growth [3]. Activated fibroblasts in the stroma, called cancer-associated fibroblasts (CAFs), interact with cancer cells and are involved in cancer progression, invasion, metastasis, and resistance to anticancer drugs [4]. Carcinogenesis of BTCs is closely associated with chronic inflammation such as cholelithiasis, cholangitis, primary sclerosing cholangitis, and pancreaticobiliary maljunction [5, 6]. Fibroblasts in the tumor microenvironment are strongly associated with the progression and metastasis of BTCs [7]. However, there are few reports on the function of CAFs in BTCs.

In scirrhous gastric cancer and pancreatic ductal adenocarcinoma (PDAC), which are characterized by abundant stroma component, CAFs secret several growth factors, including transforming growth factor-beta, hepatocyte growth factor and fibroblast growth factor [8, 9]. These cytokines from CAFs have been widely reported to promote tumor progression, and alpha-smooth muscle actin (αSMA), fibroblast activation protein alpha, and podoplanin have been reported as markers of CAFs. On the other hand, Ozemir et al. and Rhim et al. each reported that depletion of αSMA-positive CAFs promoted pancreatic cancer progression [10, 11]. More recently, Mizutani et al. reported that meflin-positive CAFs suppress cancer progression [12]. Research indicates that there are cancer-promoting CAFs and cancer-suppressive CAFs [13]. In addition, Yangngam et al. reported that Interleukin (IL)-33 acts as a tumor suppressor against cholangiocarcinoma. They reported that high IL-33 level in cancer cells and in CAFs is a good prognostic marker of survival. They revealed IL-33 inhibit cancer cell migration [14]. Most cytokines in the tumor microenvironment have a promoting effect on cancer cells, but some of them have shown a suppressive effect [10–14].

The purpose of this study was to investigate the role of CAFs and to elucidate the interaction of cancer cells and CAFs in BTCs by comparing them to PDAC cells, as a control. Furthermore, we explored the factors secreted by CAFs that suppress cancer progression.

## Materials and methods

### Patient population and tissue samples

This retrospective study includes 114 patients with PDAC and 154 patients with BTCs who underwent surgical treatment at our institution from 1996 to 2017. Clinical data and formalin fixed paraffin-embedded tissues were analyzed. BTCs include intrahepatic (*n* = 24), perihilar, and distal bile duct cancer (*n* = 67), gallbladder cancer (*n* = 27), and ampulla of Vater cancer (*n* = 36). Pathological findings were evaluated using TNM classification of the UICC guideline, eighth edition [15]. After surgical treatment, the patients were followed-up at 3–6-month intervals by clinical examinations and enhanced computed tomography. Recurrence-free survival (RFS) and overall survival (OS) were defined as the time from surgery to recurrence and death, respectively. The study was conducted in accordance with the Declaration of Helsinki and approved by the Ethics Committee of Osaka City University (approval number: 924). Written informed consent were obtained from all patients for use of tissue sample in this research.

### Immunohistochemical determination

For immunohistochemical analysis, 4-μm thick sections were obtained from the tissue microarray of formalin fixed paraffin-embedded tissues. Immunohistochemical staining was examined. The sections were deparaffinized and autoclaved for 10 min at 105 °C in Target Retrieval Solution (Dako, Carpinteria, CA, USA). After blocking the endogenous peroxidase activity, the samples were incubated with anti-human αSMA antibody (1:50, Dako, Carpinteria, CA, USA) over night at 4 °C. The sections were then incubated with biotinylated IgG for 10 min. The slides were treated with streptavidin-peroxidase reagent, followed by counterstaining with Mayer’s hematoxylin. CAFs were located around the cancer cells and stained brown. The staining intensity of spindle-shaped cells in the stroma as well as the stained area was evaluated on a 4 scale (0–3). Next, the intensity score was summed up from the staining intensity and the stained area. And then, the group of CAFs was assigned 4 scales according to the intensity score (score 1 = no staining, 2 = weak, 3 = moderate, > 4 = strong). Expression levels were considered positive when moderate or strong staining, and negative when no or week staining. Immunohistochemical evaluation was performed by two independent investigators who were blinded to patient outcomes and clinicopathological features.

### Cell line and cancer associated fibroblast

In this study, six cell lines were used: two PDAC cell lines (OCUP-A1, OCUP-A2) [16], two BTCs cell lines (OCUG, OCUCh-LM1) [17, 18], HuCCT1 purchased from RIKEN BRC (BioResource Research Center, Tsukuba, Japan), and RBE purchased from RIKEN BRC (BioResource Research Center). OCUP-A was established from anaplastic pancreatic adenocarcinoma. OCUG was established from gallbladder cancer. OCUCh-LM1 was established from a liver metastasis of extrahepatic bile duct cancer. These four OCU series cell lines were established in Department of Gastroenterological Surgery at Osaka City University Graduate School of Medicine. CAFs were obtained and isolated from specimens of pancreatic and distal bile duct cancer that underwent surgical resection at our institution from 2017 to 2019. The specimens were sliced and digested with collagenase (type I; Thermo Fisher Scientific, MA, USA) at 37 °C for 4 h. After incubation, the specimens with medium were put into a 50 ml tube through a sterile cell strainer. The cell suspensions were spined down using a centrifuge. Then the cells were collected and cultured in Dulbecco’s modified Eagle medium (DMEM; Nikken, Kyoto, Japan). To determine CAFs, immunohistochemical staining was performed. Fibroblast cells were seeded into chamber slide and fixed with methanol for 10 min. They were then incubated with anti-αSMA antibody (clone 1A4; 1:200; Dako, Cambridge, UK) for 1 h and counterstained with Mayer’s hematoxylin. Cells with aSMA-positive were determined as CAFs (Supplementary Fig. 1). All CAFs used in the experiments were at less than 10 passages. The BTC CAFs and PDAC CAFs were isolated from different patients. The culturing medium consisted of DMEM (Nikken, Kyoto, Japan) without serum. The cells were cultured at 37 °C in 21% O2 for 24 hours in 10 mL serum-free DMEM to obtain CAFs-conditioned medium (CM-CAFs).

### Cell proliferation assay

Each cell line was washed twice with phosphate-buffered saline and cultured at 5000 cells/well in 96 wells. Each cell line was incubated in 50 μL of serum-free DMEM and 50 μL of CM-CAFs for 3 days, and cell proliferation was evaluated using the CCK-8 cell counting kit (Dojindo, Kumamoto, Japan). Recombinant human IL-8, anti-human IL-8 antibody and anti-human C-X-C motif chemokine receptor 2 (CXCR2) antibody (each from R&D Systems, Minneapolis, MN, USA) were added to 100 μL of serum-free DMEM. After incubation for 3 days, cell proliferation was evaluated using the CCK-8 and MTT assay (Dojindo, Kumamoto, Japan). The control medium contained 100 μL of serum-free DMEM.

### Cytokine assay

The Human XL Cytokine Array Kit was purchased from R&D Systems (Minneapolis, MN, USA) and experiments for measuring the cytokine content of the CAFs-conditioned medium were performed according to the manufacturer’s protocols. Complete list of Human XL Cytokine Array Kit is in Supplementary Table 1.

### Western blotting

Each cancer cell was lysed on ice to collect protein. Total protein was quantified using Coomassie Plus Assay Kit (Thermo Fisher Scientific). The protein was transferred to a polyvinylidene difluoride membrane. The membranes were placed in each primary antibody: CXCR2 (1:2000, R&D Systems) or β-actin (1:5000; Sigma-Aldrich, St. Louis, MO, USA) at 4 °C overnight. The membranes were incubated with secondary antibody for 1 h and were detected by enhanced chemiluminescence using ECL prime (GE Health Care, Buckinghamshire, UK).

### Statistical analysis

Continuous variables were compared using the Mann-Whitney U test. Categorical variables were compared using chi-square or Fisher exact tests, as appropriate. OS and RFS were estimated using the Kaplan–Meier method, and survival curves were compared using the log-rank test. The groups were considered significantly different at *p* < 0.05. All tests were performed using JMP software version 13 (SAS Institute, Cary, NC, USA).

## Results

### Clinicopathological characteristics of PDAC and BTCs with high and low αSMA expression

**Fig. 1:**
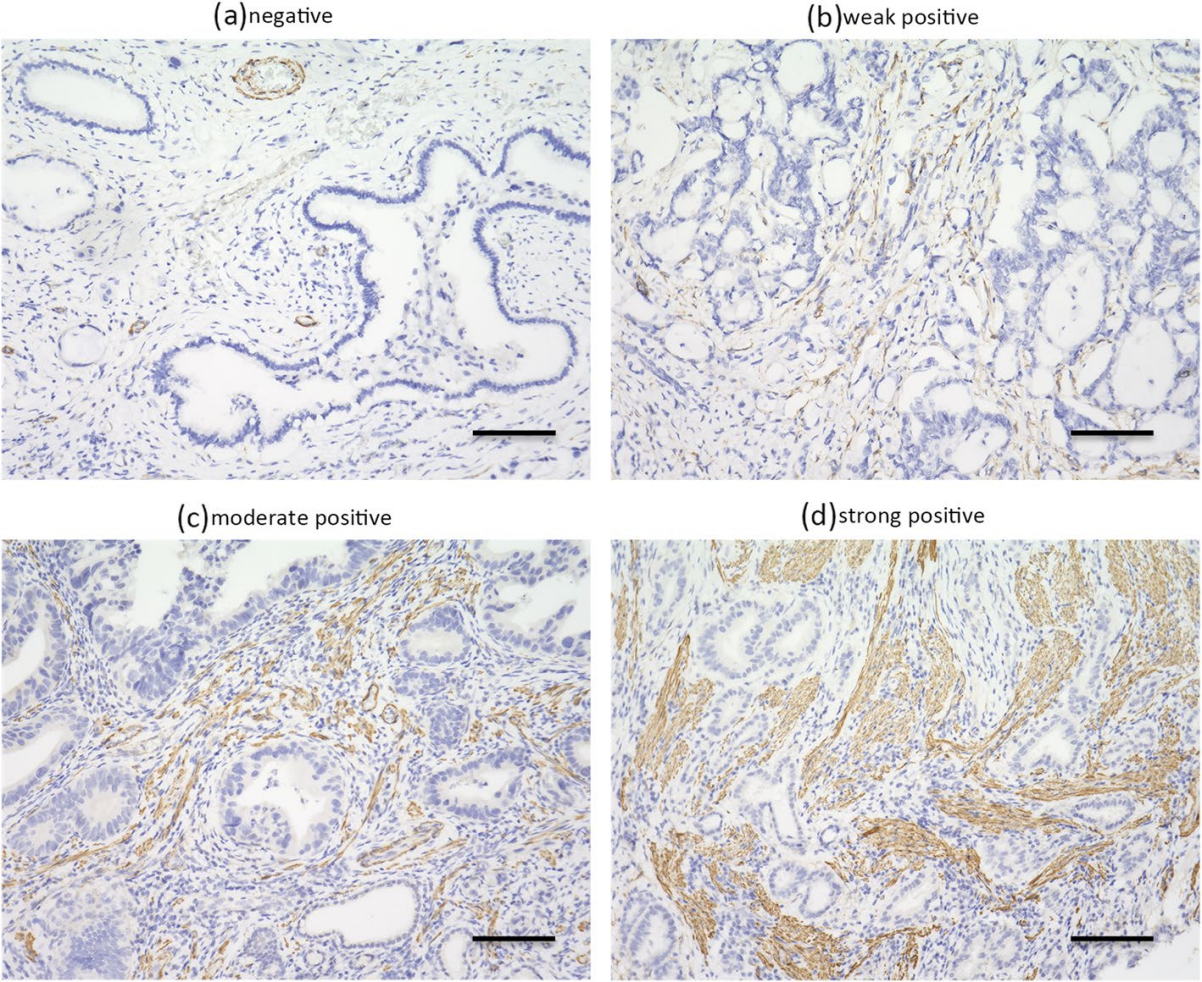
Immunohistochemical staining of αSMA. Microscopic images showing (**a**) negative, (**b**) weakly positive, (**c**) moderate positive, and (**d**) strongly positive staining. Magnification is 200x, and the scale bar is 100 μm. The αSMA-positive CAFs in the stroma are stained brown. Each patient is classified into either αSMA-positive or αSMA-negative expression group. Expressions are considered positive for scores of 2 or 3 and negative for scores of 0 or 1. αSMA: alpha-smooth muscle actin, CAFs: cancer-associated fibroblasts

The clinicopathological characteristics of the 114 resected cases of PDAC and the 154 resected cases for BTCs are listed in Tables 1 and 2. All patients were classified into an αSMA-positive or αSMA-negative expression group based on the defined criteria (Fig. 1). In patients with PDAC, positive αSMA expression was not associated with any clinicopathological characteristics (Table 3). In patients with BTCs, positive αSMA expression was associated with T category (≤pT2), absence of lymph node metastasis, absence of distant metastasis, absence of lymphatic invasion, absence of neural invasion, UICC stage (≤ Stage 2), low serum CA19–9 levels (Table 3).

**Table 1:** Clinicopathological characteristics of 114 patients with PDAC

		number
Sex	men	58
	women	56
Age, median (range)		70 (34–85)
Differentiated	differentiated	91
	undifferentiated	16
	other	7
Location of cancer	Head	68
	Body/head	46
T category	pT1	6
	pT2	23
	pT3	82
	pT4	3
Lymph node metastasis	absent	54
	present	60
Distant metastasis	absent	106
	present	8
Lymphatic invasion	absent	16
	present	98
Vascular invasion	absent	67
	present	47
Neural invasion	absent	18
	present	96
UICC stage	1	17
	2	86
	3	3
	4	8
Serum CEA level, ng/ml, median (range)		3.6 (0.5–262)
Serum CA19–9 level, U/ml, median (range)		98 (0–10,148)
Serum SPan-1 level, U/ml, median (range)		52 (1–2411)
Recurrence	yes	80
	no	34
Outcome	death	75
	alive	39
Recurrence free survival, days, median (range)		289 (0–5257)
Overall survival, days, median (range)		632 (26–1815)

*PDAC* pancreatic ductal adenocarcinoma, *UICC* Union for International Cancer Control, *CEA* carcinoembryonic antigen, *CA19–9* carbohydrate antigen 19–9, *SPan-1* s-pancreas-1 antigen

**Table 2:** Clinicopathological characteristics of 154 patients with BTCs

		number
Sex	men	86
	women	68
Age, median (range)		69 (43–87)
Differentiated	differentiated	123
	undifferentiated	14
	other	17
Location of cancer	peripheral and distal bile duct	67
	intrahepatic bile duct	24
	gallbladder	27
	ampullary	36
T category	pT0	19
	pT1	21
	pT2	49
	pT3	58
	pT4	7
Lymph node metastasis	absent	98
	present	49
Distant metastasis	absent	136
	present	11
Lymphatic invasion	absent	60
	present	64
Vascular invasion	absent	21
	present	103
Neural invasion	absent	58
	present	62
UICC stage	0	19
	1	23
	2	66
	3	42
	4	4
Serum CEA level, ng/ml, median (range)		2.6 (0–86.5)
Serum CA19–9 level, U/ml, median (range)		29 (0–45,152)
Recurrence	yes	76
	no	78
Outcome	death	70
	alive	84
Recurrence free survival, days, median (range)		521 (0–4160)
Overall survival, days, median (range)		778 (9–4157)

*BTCs* bile tract cancers, *UICC* Union for International Cancer Control, *CEA* carcinoembryonic antigen, *CA19–9*; carbohydrate antigen 19–9

**Table 3:** Correlation between clinicopathological features and αSMA in 114 patients with PDAC and in 154 patients with BTCs

		PDAC		*P* value	BTC		*P* value
		αSMA	αSMA		αSMA	αSMA	
		positive	negative		positive *n* = 83	negative *n* = 71	
		*n* = 67	*n* = 47				
Sex	men	37	21	0.26	46	40	0.26
	women	30	26		37	31	
Age, median (range)		68(34–85)	73(56–83)	0.06	70(43–87)	68(43–86)	0.23
T category	pT0–2	20	9	0.19	56	33	^a^0.008
	pT3–4	47	38		27	38	
Lymph node metastasis	absent	32	22	0.92	64	39	^a^0.003
	present	35	25		19	32	
Distant metastasis	absent	61	45	0.31	81	62	^a^0.01
	present	6	2		2	9	
Lymphatic invasion	absent	6	10	0.06	43	17	^a^0.01
	present	61	37		32	32	
Vascular invasion	absent	38	29	0.59	66	37	0.06
	present	29	18		9	12	
Neural invasion	absent	10	8	0.76	43	15	^a^0.001
	present	57	39		28	34	
UICC stage	≦2	59	44	0.31	69	41	^a^ < 0.001
	> 2	8	3		14	30	
Serum CEA level	< 5 ng/ml	38	29	0.72	63	56	0.62
	≧5 ng/ml	27	18		14	10	
Serum CA19–9 level	< 37 U/ml	19	18	0.26	52	28	^a^0.008
	≧37 U/ml	48	29		28	37	
Serum SPan-1 level	< 30 U/ml	18	20	0.09			
	≧30 U/ml	48	27				

*αSMA* alpha-smooth muscle actin, *PDAC*: pancreatic ductal adenocarcinoma, *BTCs* bile tract cancers, *UICC* Union for International Cancer Control, *CEA* carcinoembryonic antigen, *CA19–9* carbohydrate antigen 19–9, *SPan-1* s-pancreas-1 antigen

^a^*p* < 0.05

### Survival analysis

**Fig. 2:**
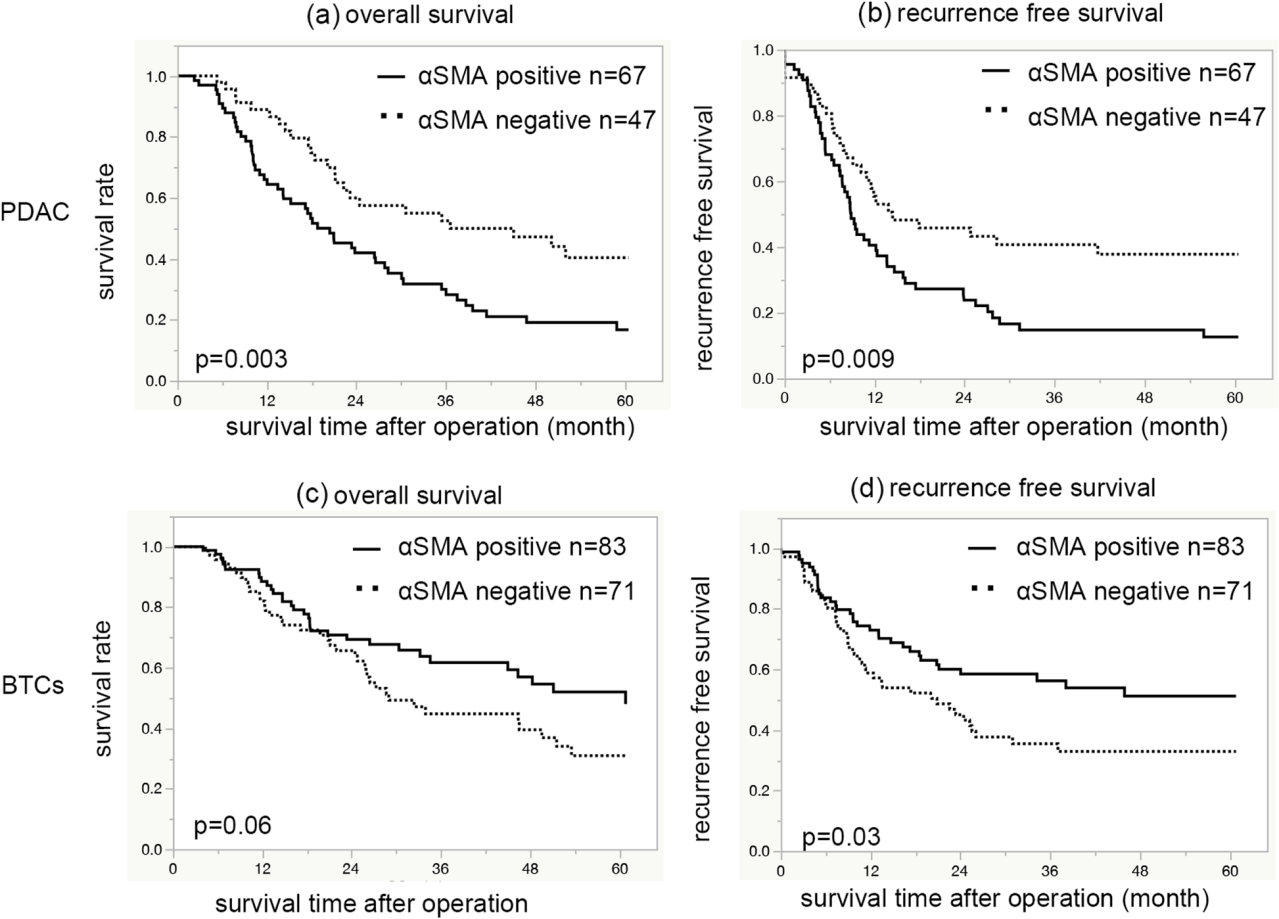
Overall survival and recurrence-free survival according to αSMA in PDAC and BTCs. Kaplan-Meier survival curve indicates that αSMA-positive PDAC patients show significantly shorter OS than those with αSMA-negative expression (**a**), with similar results for RFS (**b**). **c** Patients with αSMA-positive expression tend to have better OS compared to those with αSMA-negative expression in BTCs. **d** BTC patients with αSMA-positive expression show significantly better RFS compared to those with αSMA-negative expression. αSMA: alpha-smooth muscle actin, PDAC: pancreatic ductal adenocarcinoma, BTCs: biliary tract cancers, OS: overall survival, RFS: recurrence-free survival

For patients with PDAC, those with positive αSMA expression showed significantly shorter OS than those with negative αSMA expression (median OS, 20.4 vs. 36.6 months; 5-year survival rate, 14.7 vs. 39.2%, *p* = 0.003) (Fig. 2a). Similarly, the αSMA-positive group showed statistically shorter RFS, compared to the αSMA-negative group (median RFS, 8.8 vs. 14.4 months; 5-year RFS rate, 5.8 vs. 29.9%, *p* = 0.009) (Fig. 2b). On the other hand, in the patients with BTCs, the αSMA-positive group showed better RFS compared to the αSMA-negative group (median RFS: not reached vs 20.8 months; 5-year RFS rate: 39.9% vs 19.3%, *p =* 0.03) (Fig. 2d). In the OS of patients with BTCs, the αSMA-positive group tended to have better OS compared to the αSMA-negative group (median OS: 60.8 vs 29 months; 5-year survival rate: 47.4% vs 31.5%, *p =* 0.06) (Fig. 2c).

### Effect of CM-CAFs on cancer cell proliferation

**Fig. 3:**
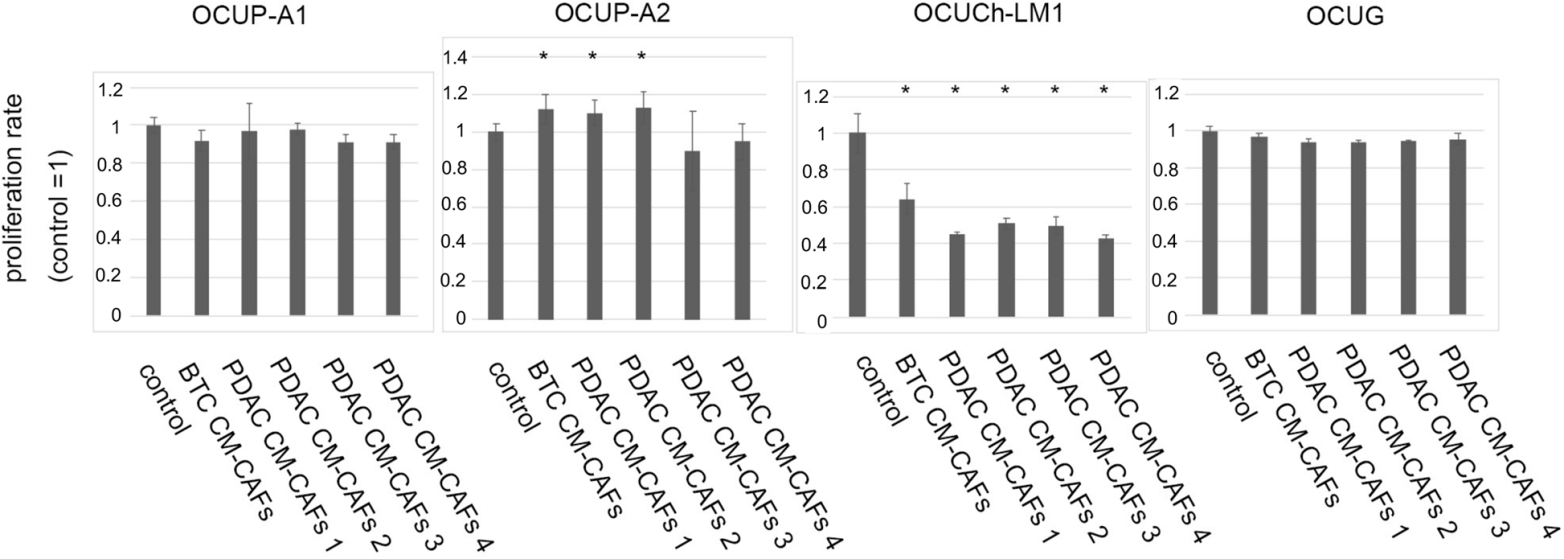
Effect of CM-CAFs on proliferation of each cell line. Conditioned medium from each CAFs (CM-CAFs) significantly promote proliferation of OCUP-A2 cells but significantly suppress proliferation of OCUCh-LM1. Data are presented as the mean and standard deviation of four experiments. Asterisks indicate a significant difference. *p < 0.05. CAFs: cancer-associated fibroblasts

In order to evaluate the effect of CM-CAFs on cancer cell proliferation, CM-CAFs from PDAC or BTC was added to each cell line (OCUP-A1, OCUP-A2, OCUG and OCUCh-LM1). Although none of the CM-CAFs affected OCUP-A1 or OCUG cell proliferation, the BTC CM-CAFs and PDAC CM-CAFs 1 and 2 promoted cell proliferation compared to OCUP-A2 growth in the control medium. On the other hand, all CM-CAFs significantly suppressed the proliferation of OCUCh-LM1 compared to growth in the control medium (Fig. 3).

### Cytokines contained in CM-CAFs with inhibition effect

**Fig. 4:**
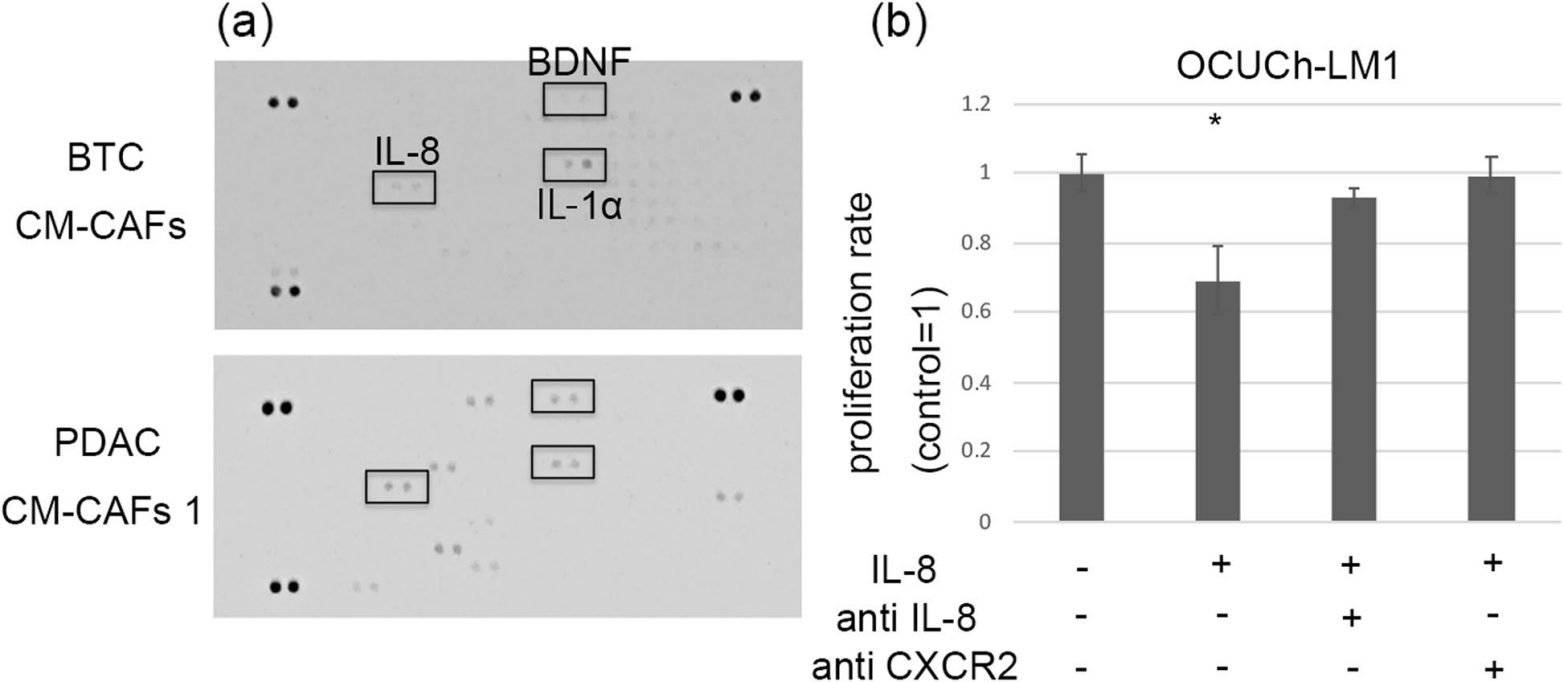
Cytokine array of CM-CAFs and effect of IL-8 on proliferation of OCUCh-LM1 cells.** a** Cytokine array compares BTC CM-CAFs and PDAC CM-CAFs 1. BDNF, IL-8, and IL-1α are commonly included. **b** IL-8 significantly suppresses proliferation of OCUCh-LM1 cells. Addition of antibodies against IL-8 or CXCR2 alleviates the suppression effect of IL-8. Each concentration of antibody is IL-8; 100 pg/100 μL, anti IL-8; 1000 pg/100 μL, anti CXCR2; 1000 pg/100 μL, respectively. Data are presented as the mean and standard deviation of four experiments. Asterisks indicate a significant difference from the control medium. *p < 0.05. CM-CAFs: Conditioned medium from cancer-associated fibroblasts, IL-8: Interleukin-8, BTCs: biliary tract cancers, PDAC: pancreatic ductal adenocarcinoma, IL-1α: Interleukin-1α, BDNF: brain-derived neurotrophic factor, CXCR: C-X-C motif chemokine receptor

To determine the cytokine content of the CAFs that had a suppression effect, cytokine assays were performed on BTC CM-CAFs and PDAC CAFs 1 which had suppressed OCUCh-LM1 cell proliferation. The cytokines commonly included were IL-8, IL-1α, and brain-derived neurotrophic factor (BDNF) (Fig. 4a). For OCUCh-LM1, the addition of IL-8 had a suppressive effect on proliferation. The addition of IL-1α promoted OCUCh-LM1 cell proliferation. The addition of BDNF had no effect on OCUCh-LM1 cell proliferation (Supplementary Fig. 2). The addition of IL-8 did not affect cancer cell proliferation for OCUP-A1, OCUP-A2, OCUG, HuCCT-1, or RBE (Supplementary Fig. 3). Both CCK and MTT assays showed similar IL-8 suppressive effects on OCUCh-LM1 cells.

### Effect of IL-8 on cell proliferation of OCUCh-LM1

To confirm the suppressive effect of IL-8 on OCUCh-LM1 cell proliferation, anti-human IL-8 antibody was added to the cell culture medium. After the addition of anti-human IL-8 antibody, the suppressive effect of IL-8 disappeared. Anti-human CXCR2 antibody suppressed cell growth similar to that of the anti-human IL-8 antibody (Fig. 4b).

### Expression of CXCR2 on cancer cell lines

Supplementary Fig. 4 showed that CXCR2 was expressed on all cell lines, but the expression level was highest on OCUCh-LM1.

## Discussion

In this study, we found that CAFs that express αSMA are a poor prognostic factor in patients with PDAC. On the other hands, CAFs in BTCs were a good prognostic factor. In addition, we demonstrated in vitro that IL-8 produced from CAFs suppresses the proliferation of OCUCh-LM1 cells. Previous reports have indicated that CAFs promote cancer growth by interacting with cancer cells but are a poor prognostic factor in several cancer types [19, 20]. CAFs often affect cancer progression by interacting with cancer cells via cytokines and exosomes [8, 9]. The current result suggested that CAFs have a one-sided effect on suppressing cancer progression in BTCs.

αSMA is the best-known marker for CAFs and has been identified as a poor prognostic factor in several cancers [21]. Akatsu et al. reported that αSMA-positive CAFs, called type II CAFs, are associated with the endothelial-to-mesenchymal transition and promote tumor growth and metastasis [22]. Augsten reported that cancer-suppressive CAFs, type I CAFs, do not express αSMA, have the ligand Slit2, and inhibit the tumorigenicity of cancer cells [23]. The current study suggests that αSMA expression in CAFs is a good prognostic factor in BTCs, but not in PDAC. This is the first report of αSMA-positive CAFs in BTCs being a good prognostic factor.

We hypothesized that there were molecules, especially the cytokines secreted by αSMA-positive CAFs, which might have a suppressive effect on the proliferation of OCUCh-LM1 cells. The results show that CM-CAFs, which wither promoted proliferation or had no effect in PDAC cells, showed a suppressive effect on OCUCh-LM1 cell proliferation. More interestingly, we found that both BTC CM-CAFs and PDAC CM-CAFs contained factors that have suppressive effects on OCUCh-LM1 cell proliferation. We previously reported cytokines and exosomes are found in CM-CAFs [8, 24]. In the current study, we demonstrated that IL-8 secreted by CAFs suppresses OCUCh-LM1 cell proliferation.

The most interesting finding in this study was that IL-8 produced from the CM-CAFs of PDAC and BTC suppressed the proliferation of OCUCh-LM1 cells. Although IL-8 is well known as inflammatory cytokines [25], there are many reports of chemokines from CAFs that promote the proliferation and migration of cancer cells [26, 27]. The function of IL-8 depends on its interaction with its receptors, CXCR1 and CXCR2. The CXCR1 receptors are activated only in response to binding of IL-8, whereas CXCR2 receptors are activated by several chemokines [28]. Wang et al. reported that CXCR1 expression correlates with drug resistance, invasion and metastasis in many types of cancers [29]. On the other hand, IL-8 and CXCR2 are also involved in cell proliferation and cell senescence. CXCR2 is upregulated during senescence [30, 31]. We also reported that CXCL1-CXCR2 signaling have tumor suppression roles in cholangiocarcinoma [32]. Therefore, it may be that CXCR-2 is more involved than CXCR1 in cancer suppression. Thus, we investigated IL-8/CXCR2 signaling. Here, we found that IL-8 produced from CAFs suppresses the proliferation of OCUCh-LM1 cell line. In addition, we demonstrated that the addition of the antibodies that inhibit IL-8 or CXCR2 eliminated their suppressive effect. Therefore, we suggest that IL-8/CXCR2 signaling pathway might be a mechanism that suppresses OCUCh-LM1 growth. However, CXCR2 expression was observed in each cell line suggesting that even with the same receptor and signaling, the functions of IL-8/CXCR2 signaling might be changed depending on the difference in expression level of CXCR2 and the characteristics of the cancer itself. In addition, the effect of IL-8/CXCR2 signaling on cell senescence is needed in the future.

There have been no reports of αSMA-positive CAF in biliary tract cancer as a good prognosis factor of survival. This study revealed that anti-IL-8 antibody and anti-CXCR2 antibody are able to inhibit the suppressive effects of IL-8. However, these antibodies were not able to inhibit the suppressive effects of CM-CAFs (data not shown). This result indicated that the suppressive effect came from not only IL-8 alone, but also several cytokines produced by CAF. Also, the balance of chemokines and the expression level of receptors were affected. Therefore, further explorations are needed to achieve therapeutic development.

This study has limitations. First, this was a retrospective study with a small cohort of patients. Second, the number of CAFs and cell lines was low and limited. Third, CAFs had a mixed population with αSMA positive and negative fibroblasts, therefore, it was unclear which type of CAFs had suppressive effects on OCUCh-LM1 cell proliferation. Fourth, Human XL Cytokine Array Kit could investigate only 105 typical cytokines, so other cytokines might have been overlooked. Lastly, it is difficult to establish and passage CAFs from biliary tract cancer and pancreatic cancer. Therefore, it is also difficult to repeatedly experiment with the same CAFs.

In summary, this study suggests that CAFs are a good prognostic factor in patients with BTCs, but not in those with PDAC. This is the first report of αSMA-positive CAFs in BTCs being a good prognostic factor of survival. In addition, IL-8 found in CM-CAFs suppresses the proliferation of OCUCh-LM1 cells. Our findings suggest that CAFs have tumor-suppressive activity in BTCs via their own humoral factors, including IL-8.

## Supplementary Information

**Additional file 1.**

**Additional file 2.**

**Additional file 3.**

**Additional file 4.**

**Additional file 5.**

## Data Availability

The datasets used and analyzed during the current study are available from the corresponding author on reasonable request.

## Abbreviations

CAFs: cancer-associated fibroblasts
BTCs: biliary tract cancers
PDAC: pancreatic ductal adenocarcinoma
αSMA: alpha-smooth muscle actin
IL-8: Interleukin-8
UICC: Union for International Cancer Control
RFS: recurrence-free survival
OS: overall survival
DMEM: Dulbecco’s modified Eagle medium
CM: conditioned medium
CCK: cell counting kit
CXCR: C-X-C motif chemokine receptor
CEA: carcinoembryonic antigen
CA19–9: carbohydrate antigen 19–9
SPan-1: s-pancreas-1 antigen
IL-1α: Interleukin-1α
BDNF: brain-derived neurotrophic factor
