# Supplementary figures and images for: Interleukin-8 produced from cancer-associated fibroblasts suppresses proliferation of the OCUCh-LM1 cancer cell line
Source: BMC Cancer. 2022 Jul 8;22:748. doi: 10.1186/s12885-022-09847-z (PMC9270823; doi:10.1186/s12885-022-09847-z)

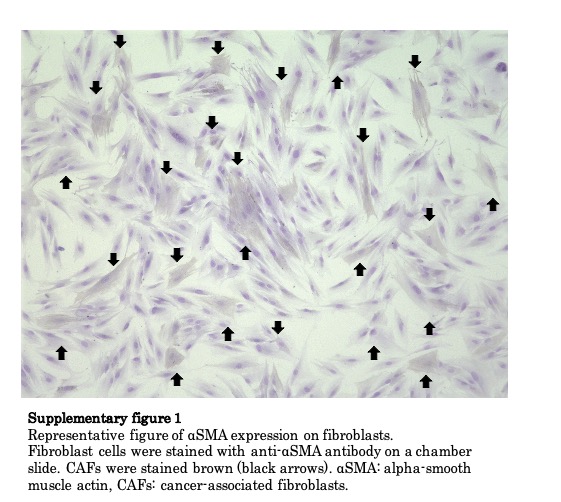

Supplement: Supplementary file 1 — Additional file 1. [file 12885_2022_9847_MOESM1_ESM.jpg]

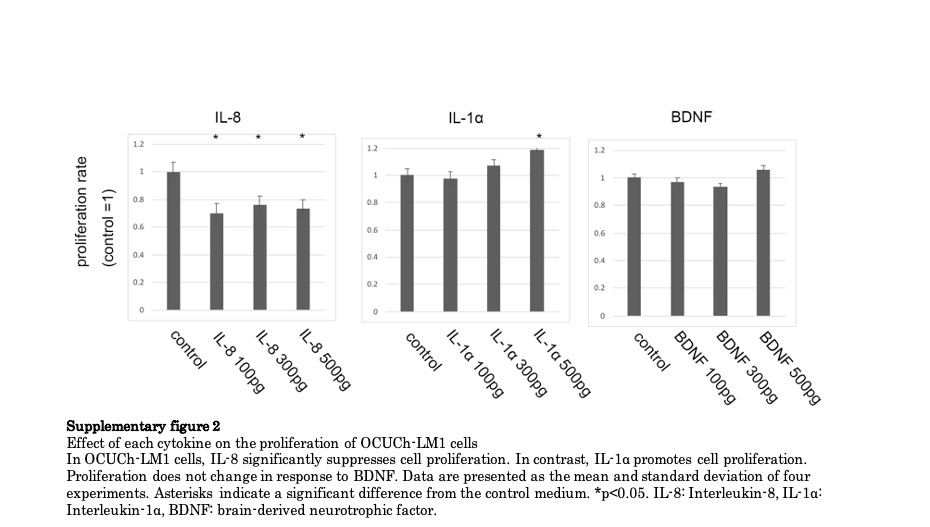

Supplement: Supplementary file 3 — Additional file 3. [file 12885_2022_9847_MOESM3_ESM.jpg]

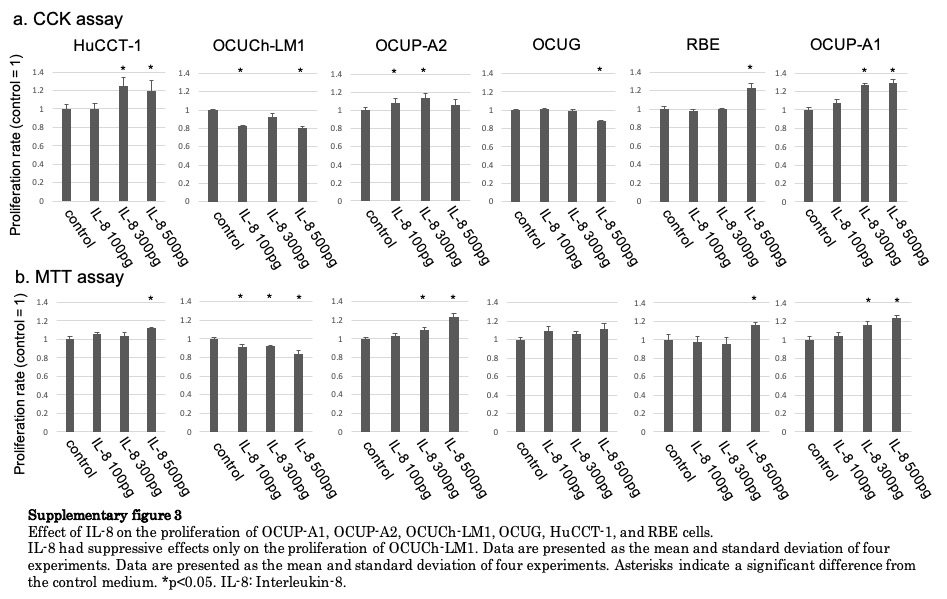

Supplement: Supplementary file 4 — Additional file 4. [file 12885_2022_9847_MOESM4_ESM.jpg]
