# Supplementary material for: Interleukin-8 produced from cancer-associated fibroblasts suppresses proliferation of the OCUCh-LM1 cancer cell line
Source: BMC Cancer. 2022 Jul 8;22:748. doi: 10.1186/s12885-022-09847-z (PMC9270823; doi:10.1186/s12885-022-09847-z)
Supplement: Supplementary file 2 — Additional file 2. [file 12885_2022_9847_MOESM2_ESM.docx]

Supplementary table 1. Complete list of Human XL Cytokine Array Kit

| Adiponectin | IGFBP-2 | MCP-3 |
| --- | --- | --- |
| Apolipoprotein A-I | IGFBP3 | M-CSF |
| Angiogenin | IL-1α | MIF |
| Angiopoietin-1 | IL-1β | MIG |
| Angiopoietin-2 | IL-1ra | MIP-1α/MIP-1β |
| BAFF | IL-2 | MIP-3α |
| BDNF | IL-3 | MIP-3β |
| Complement Component C5/C5a | IL-4 | MMP-9 |
| CD14 | IL-5 | Myeloperoxidase |
| CD30 | IL-6 | Osteopontin |
| CD40 ligand | IL-8 | PDGF-AA |
| Chitinase 3-like 1 | IL-10 | PDGF-AB/BB |
| Complement Factor D | IL-11 | Pentraxin 3 |
| C-Reactive Protein | IL-12 p70 | PF4 |
| Cripto-1 | IL-13 | RAGE |
| Cystatin C | IL-15 | RANTES |
| Dkk-1 | IL-16 | RBP-4 |
| DPPIV | IL-17A | Relaxin-2 |
| EGF | IL-18 Bpa | Resistin |
| EMMPRIN | IL-19 | SDF-1α |
| ENA-78 | IL-22 | Serpin E1 |
| Endoglin | IL-23 | SHBG |
| Fas Ligand | IL-24 | ST2 |
| FGF basic | IL-27 | TARC |
| FGF-7 | IL-31 | TFF3 |
| FGF-19 | IL-32 | TfR |
| Flt-3 Ligand | IL-33 | TGF-α |
| G-CSF | IL-34 | Thrombospondin-1 |
| GDF-15 | IP-10 | TNF-α |
| GM-CSF | I-TAC | uPAR |
| GR0α | Kallikrein 3 | VEGF |
| Growth Hormone | Leptin | Vitamin D BP |
| HGF | LIF | TIM-3 |
| ICAM-1 | Lipocalin-2 | VCAM-1 |
| IFN-γ | MCP-1 | Negative Control |
