# Supplementary material for: Interleukin-8 produced from cancer-associated fibroblasts suppresses proliferation of the OCUCh-LM1 cancer cell line
Source: BMC Cancer. 2022 Jul 8;22:748. doi: 10.1186/s12885-022-09847-z (PMC9270823; doi:10.1186/s12885-022-09847-z)
Supplement: Supplementary file 5 — Additional file 5. [file 12885_2022_9847_MOESM5_ESM.pdf]

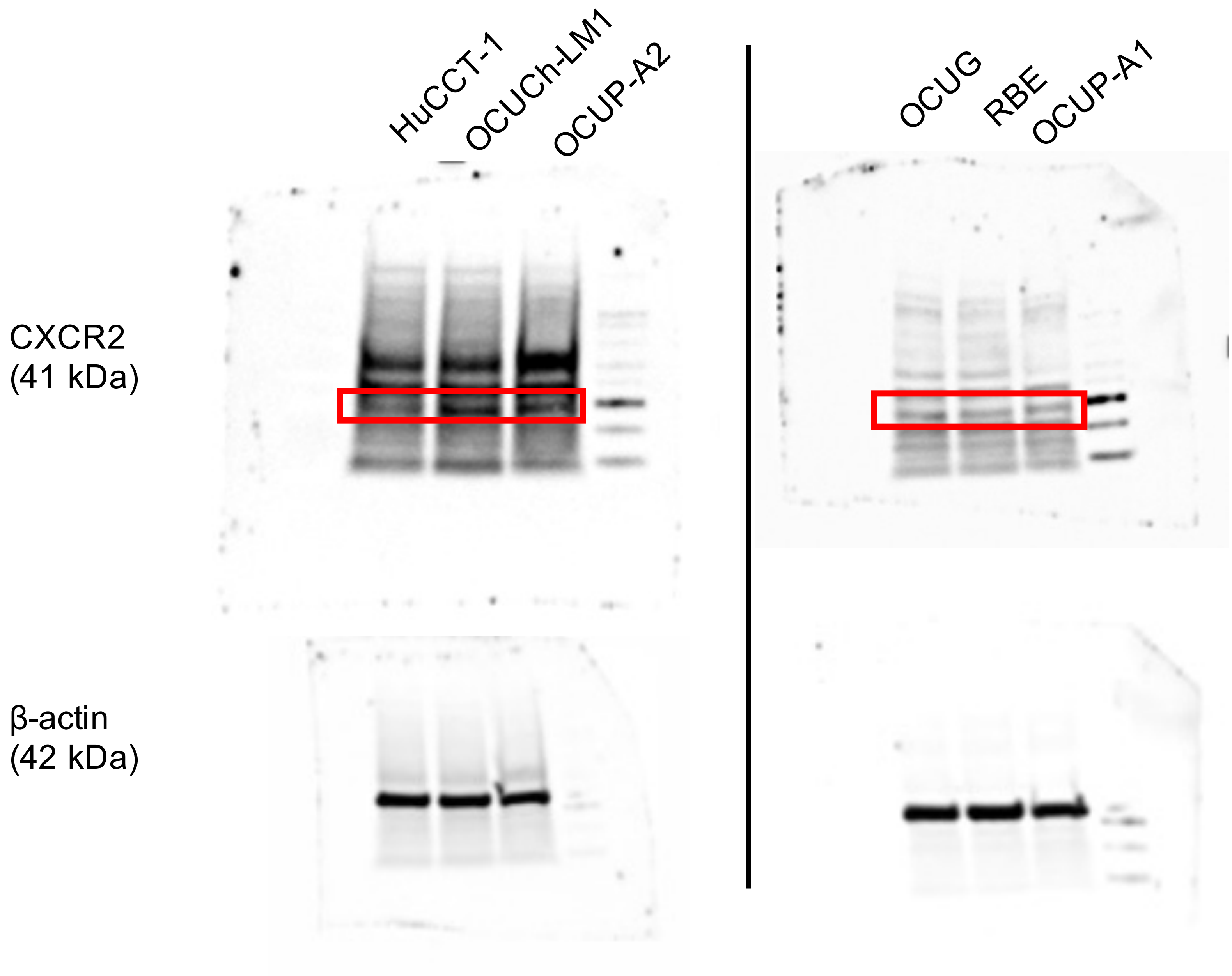

Supplementary figure 4

CXCR2 expression on HuCCT-1, OCUCh-LM1, OCUP-A2, OCUG, RBE, and OCUP-A1. CXCR2 expression level was highest on OCUCh-LM1. These samples were collected with the same procedure and that gels/blots were processed in parallel. Loading controls was done on the same blot. CXCR2: C-X-C motif chemokine receptor 2.
